# Spectroscopic and Electrochemical Exploration of Carbon-Infused Intercalation-Type Spinel Composite for Aqueous Systems

**DOI:** 10.3389/fchem.2022.890291

**Published:** 2022-07-13

**Authors:** Shane Willenberg, Emanuela Carleschi, Natasha Ross

**Affiliations:** ^1^ Sensorlab, University of the Western Cape, Bellville, South Africa; ^2^ Department of Physics, Auckland Park, University of Johannesburg, Johannesburg, South Africa

**Keywords:** intercalation material, aqueous system, MWCNTs, charge transfer, spectroscopy, electrochemistry: XPS

## Abstract

Lithium-manganese-based compounds are promising intercalation host materials for aqueous battery systems due to their synergy with high ionic conductive aqueous electrolytes, safety, eco-friendliness, and low cost. Yet, due to poor electrical conductivity and trapping of diffused electrolyte cations within its crystal formation, achieving optimum cycle stability and rate capability remains a challenge. This unfortunately limits their use in modern day high-powered devices, which require quality output with high reliability. Here, the authors propose a facile method to produce LiMn_2_O_4_ and LiFe_0.5_Mn_0.5_PO_4_ and compare their structural stability and corresponding electrochemical performance by controlling the interfacial layer through multi-walled carbon nanotubes’ (MWCNTs) infusion. High-resolution scanning electron microscopy results revealed that the active particles were connected by MWCNT *via* the formation of a three-dimensional wiring network, suggesting that stronger interfacial bonding exists within the composite. As a result, the conducting composite decreases the electron transport distance with an increased number of active sites, thus accelerating the lithium ion intercalation/de-intercalation process. Compared to C/LMO with a R_ct_ of 226.3 Ω and change transfer (i_o_) of 2.75 × 10^−3^, the C/LFMPO**-**composite has a reduced R_ct_ of 138 Ω and enhanced rate of 1.86 × 10^−4^ A cm^−2^. The faster kinetics can be attributed to the unique synergy between the conductive MWCNTs and the contribution of both single-phase and two-phase regions in Li_1-x_(Fe,Mn)PO_4_ during Li^+^ extraction and insertion. The electrochemical features before and after modification correlate well with the interplanar distance of the expanded manganese and manganese phosphate layers shown by their unique surface features, as analyzed by advanced spectroscopy techniques. The results reveal that MWCNTs facilitate faster electron transmission whilst maintaining the stability of the host framework, which makes them favorable as next generation cathode materials.

## Introduction

In the past decade, the energy density of current commercial batteries has been pushed to the limit, and with that has come an increase in safety issues. Lithium Manganese Oxide (LMO) and Lithium Manganese Phosphate (LMP) have both been identified as promising aqueous-type intercalation host materials for new age technological applications. This is due to their high-energy densities, rechargeability, and compact and light-weight nature, making them extremely consumer friendly (Xinga et al., 2018; Ma et al., 2022). Aqueous-based electrochemical systems are emerging as strong contenders for non-aqueous systems with distinct advantages (Willenberg and Ross, 2020) such as cost efficiency, non-toxicity, and stability at high temperature. The stable voltage window of aqueous electrolytes is, however, restricted to <1.5 V due to the slim potential window of water. This is significantly less than the value of 3 V for nonaqueous electrolytes (Dushina et al., 2015). However, for spinel-LMO, the manganese dissolution into the electrolyte is a major factor which requires chemical and physical interventions (Zhang et al., 2017). As a result, fewer lithium ions are available during charge/discharge processes and that significantly lowers the electrode capacities. Previous works have shown that the use of nanostructured LMO and modification with highly conductive agents such as graphene has the potential to significantly enhance the rate performance of LMO (Tang et al., 2015).

The LMP, on the other hand, is isostructural with triphylite and a redox potential of 4.1 V versus Li/Li^+^ with reversible topotactic Li-ion extraction, cyclability, and exceptional thermal stability. Concomitantly, this material also suffers from impaired electrochemical activity and slow lithium ion diffusion due to the Jahn−Teller active Mn^3+^ ions (Wang et al., 2018). The lithium extraction–insertion is described by the following equations:

$$LiMn_{2}O_{4}\;↔\;Li_{1-x}Mn_{2}O_{4}+x\;\cdot \;Li^{+}+x\cdot e^{-}$$ (1)

$$LiMPO_{4}-xLi^{+}-xe^{-}\;↔\left(1-x\right)LiMnPO_{4}+xMPO_{4}$$ (2)

where *x* represents the molar fraction of ions conveyed into the electrolyte during the reduction and oxidation reaction.

Through revolutionary electrochemical developments made in aqueous battery systems over the years, scientists are now able to meet consumer demands for increased safety with superior performing portable electronics and electric vehicles (Xu et al., 2020). The exploration of functionalization strategies has shown to not only significantly influence the fundamentals to mitigate the causes of capacity fading, but also provide more diverse materials with potentially better cyclability (Xu et al., 2015). In an earlier study, LMP was doped with Fe which enhanced its conductivity significantly (Sifuba et al., 2021). This is due to the collective Mn−O−Fe interactions (*via* P_tet_−O−M_oct_) generated in olivine lattices (Paolella et al., 2014) which set the redox energy of Mn^2+^/Mn^3+^ higher than that of Fe^2+/^Fe^3+^. Additionally, a considerable amount of Li^+^ (de) intercalates in the Li_1-x_(Fe,Mn)PO_4_ lattice *via* a solid solution mechanism, thus enhancing the specific power (rate capability) (Drozhzhin et al., 2016). Here, the presence of Mn^2+^ increases both the capacity and energy density compared to pure LMP, and has therefore become a hot research topic. Despite these advantages, LFMPO is still sensitive to moisture in addition to the problem of manganese dissolution (Mallick et al., 2021). To remedy these issues and improve its intrinsic conductively, literature suggests that modification with carbon to be a viable option (Chen et al., 2022). The carbon architecture facilitates effective contact between particles and reduces the Li^+^ diffusion channel in LFMPO. Thus, in this study the synergy of both LMO and LFMPO cathode materials with MWCNT was probed and compared using microscopic and spectroscopic techniques. MWCNT was the preferred carbon source due to its superb conductivity and ability to provide the required mechanical and chemical stability to improve the electrochemical performance of hybrid cathode materials in aqueous media (Yang et al., 2019).

This work focusses on establishing which intercalation host materials delivers improved electrochemical performance when functionalized with MWCNTs towards development of a stable and conductive material with reduced manganese dissolution (Dai, 2002). The carbon support is a critical component in C/LFMP and C/LMPO as it provides a comprehensive understanding of its effects on the electrochemistry of the material in aqueous media. The behavior of carbon-supported LiFe_0.5_Mn_0.5_PO_4_ cathodes in aqueous solutions has not been cited before. Here we show that the layered network of C/LiFe_0.5_Mn_0.5_PO_4_ can provide increased space for expansion upon Li-ion insertion and its zero-band gap nature can improve the electrochemical properties of the cathode, even in an aqueous system. This study may be beneficial towards accelerating the commercialization of aqueous intercalation-type electrode materials in more consumer electronics. Additionally, these water-based cathodes could improve the eco-balance of energy storage devices by omitting the use of toxic solvents (Du et al., 2011; Doeff, 2013; Biwei, 2020).

## Materials and Methods

### Apparatus for Materials’ Characterization

The nanomaterial morphology, particle size, and size distribution were probed through Scanning Electron Microscopy [SEM; JOEL JSM-7500F Scanning Electron Microscope (United States)], Transmission Electron Microscopy (TEM; Tecnai G2 F20X-Twin MAT 200 kV Field Emission Transmission Electron Microscope), and Small-angle X-ray scattering (SAXS, FEI Eindhoven, Netherlands). The Small Angle X-rays Scattering analysis was acquired from Anton Paar GmbH (Anton-Paar Str 20 A-8054 Graz). The X-ray diffraction (XRD) patterns were recorded on a Rigaku Smart Lab 3 kW diffractometer with Cu Kα radiation (*λ* = 1.5418 Å), with the corresponding operation voltage and current at 40 kV and 100 mA, respectively. All electrochemical characterizations were executed using a PalmSens devise (PSTrace 5.2). The analysis was done using a three-electrode system consisting of a glassy carbon electrode (GCE, working electrode) (d = 3 mm), platinum wire (counter electrode), and Ag/AgCl (reference electrodes) (3 M KCl). X-ray Photoemission Spectroscopy (XPS) measurements were performed on *ex-situ* powder samples mounted on the sample holder by means of carbon tape at room temperature. A SPECS XR 50M monochromatized X-ray source (Al *K*_α_ = 1,486.71 eV) and a SPECS Phoibos 150 hemispherical electron energy analyzer were used. Charge compensation was achieved by means of a low electron energy flood gun operating at an electron energy of 2.5 eV and an electron flux of 20 µA.

### Purification of Multi-Walled Carbon Nanotubes

MWCNT was baked to 300°C in a muffle furnace for 1 h before treatment with 1 M HNO_3_ solution to remove impurities. The suspension was placed under ultrasonication for 2 h. After settling at the bottom of the flask, the suspension was centrifuged and rinsed with distilled water before drying at 60°C all-night.

### Synthesis of Pure Lithium Manganese Oxide and LFMPO Intercalation Materials

The precursor spinel LMO was prepared following a previously reported method (Xinga et al., 2018) with minor modifications. In brief, stoichiometric amounts of LiOH and C_4_H_14_MnO_8_ (Li/Mn = 1:2) were dissolved in deionized water followed by gentle stirring. The precursor powder was obtained after evaporation of the solution at 120°C for 12 h. The precursor was subjected to further heat treatment at 400°C for 1 h, and then calcinated at 800°C for 20 h in a muffle furnace to form LMO.

The LFMPO composite was synthesized with the complexing agent (Ma et al., 2022) using a simple and facile microwave-assisted process (Hou et al., 2020). In brief, a 1.5 M aqueous solution of LiOH.H_2_O was added to a solution of H_3_PO_4_ + 0.5 M (NH_4_) H_2_PO_4_ and mixed by magnetic stirring at room temperature for 5 min. Thereafter, 0.5 M MnSO_4_.H_2_O, 0.5 M FeSO_4._7H_2_O, and 1 M acetic acid (CH_3_COOH) were added into the starting mixture. The mixed solution was deposited in the 100 ml XQ quartz vessel, sealed, and placed in the microwave reaction system (Multiwave PRO), which was powered at 400 W to heat the mixture for 30 min. To remove all the excess H_3_PO_4_, the final product (LFMPO) was rinsed with acetone and distilled water, centrifuged, and dried in a convention oven at 70°C overnight. To provide a uniform and crystal structure the mixture was sintered at 600°C for 6 h under Ar-H_2_ (95/5 vol%) atmosphere.

### Synthesis of C/LMO and C/LFMP Nano-Composites

**FIGURE 1:**
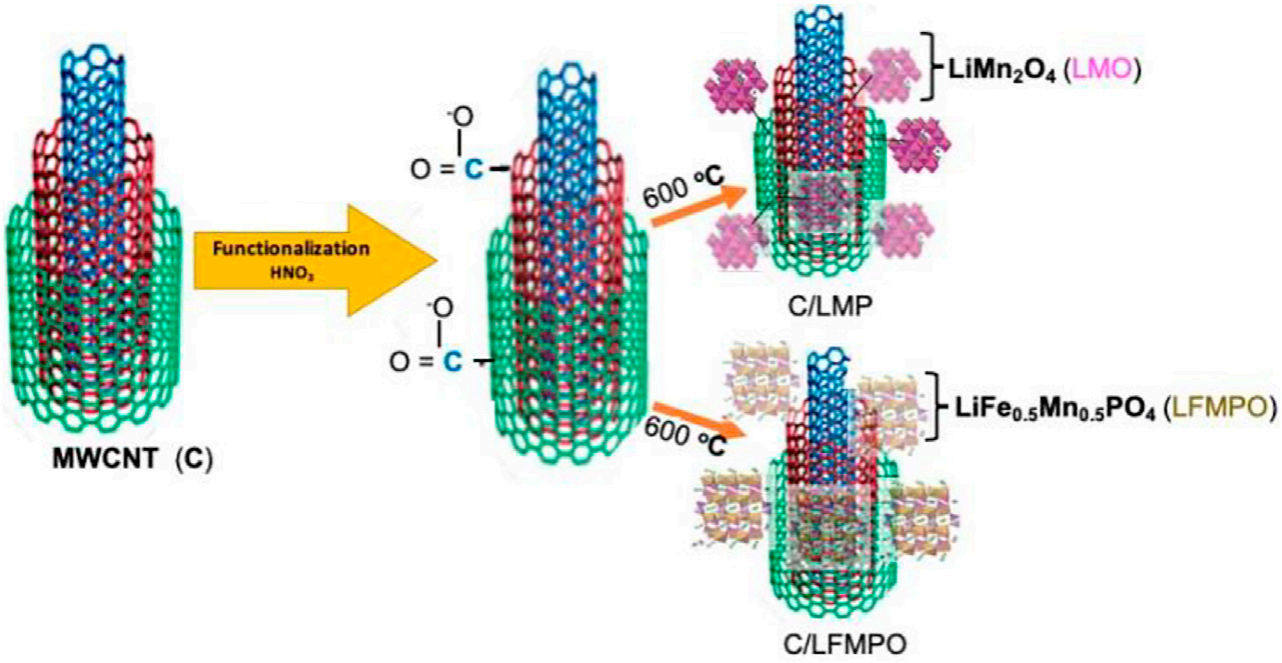
Functionalization towards formation of C/LMO and C/LFMPO.

The C/LMO and C/LFMPO was obtained after calcination with the purified MWCNT at 600 °C as depicted in Figure 1. The process involved mixing 2 mg of the activated multi-walled carbon nanotubes to both LMO and LFMPO respectively (Willenberg and Ross, 2020). The molar ratio was kept at 4:1. After adding MWCNTs and treating the mixture in an ultrasonic bath for 10 min with appropriate washing steps, the mixture was deposited in the 100 ml XQ quartz vessel, sealed, and placed in the microwave reaction system for 30 min at 400 W (Multi-wave PRO Microwave). Further heat treatment of the mixture/s produced the complete conversion into C/LMO and C/LFMPO, as confirmed by the XRD pattern in Figure 4.

### Chemicals

Analytical grade Lithium sulphate (Li2SO4, 99.9%), Lithium hydroxide monohydrate (LiOH · H_2_O, 99.995%) trace metal basis, Manganese (II) Sulfate monohydrate (MnSO_4_ · H_2_O), Diethylene glycol ((HOCH₂CH₂)₂O, 99.0%), Magnesium acetate tetrahydrate (Mg(CH_3_COO)2 . 4H_2_O, ≥ 99%), N-methyl-2-pyrrolidone, anhydrous (C5H9NO, 99.5%), Phosphoric acid (H_3_PO_4_) (85%) trace metal basis, Ammonium dihydrogen phosphate (NH_4_) (H_2_PO_4_), Lithium manganese oxide (LiMn_2_O_4_), manganese acetate tetrahydrate (C_4_H_14_MnO_8_, Aldrich), sodium borohydrate (NaBH_4_), potassium permanganate (K_2_MnO_4_), graphite (Aldrich), N-methyl-2-pyrrolidinone (NMP), hydrogenfluoride (HF), and lithium hydroxide (LiOH) were all purchased from Aldrich and Fluka, respectively, and used as received. A 1 M LiNO_3_ electrolyte solution, 0.5 M Iron (II) Sulphate Heptahydrate (FeSO_4._7H_2_O), and 1 M acetic acid were prepared and the NMP served as the binding agent in preparation of the active GCE setup. The multi-walled carbon nanotubes (MWCNTs) were commercially purchased from Sigma.

## Results and Discussion

### High Resolution Scanning Electron Microscopy and Transmission Electron Microscopy Analysis

**FIGURE 2:**
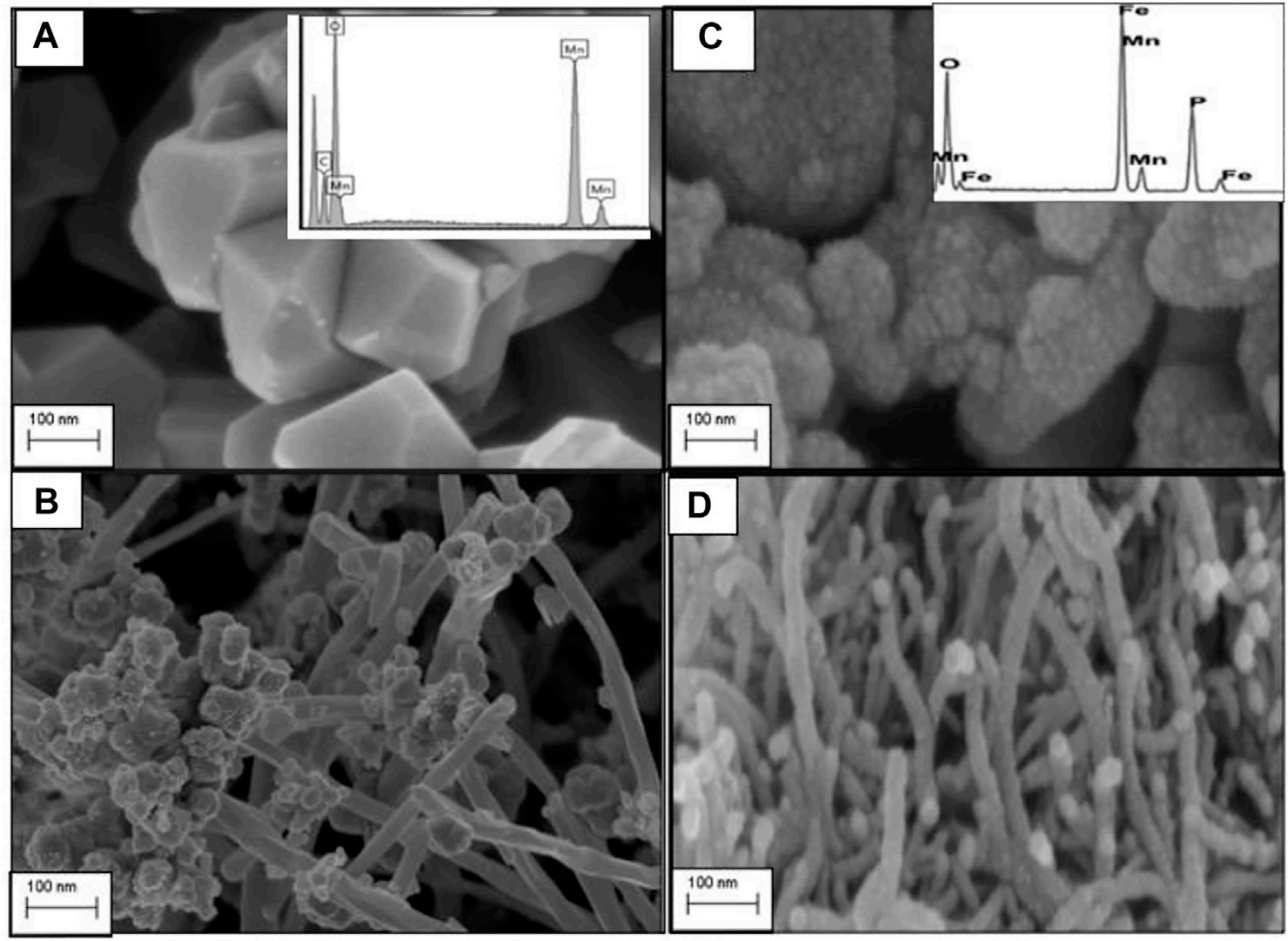
High-resolution scanning electron microscopy (HRSEM) of **(A)** LMO, **(B)** C/LMO, **(C)** LFMPO, and **(D)** C/LFMPO.

**FIGURE 3:**
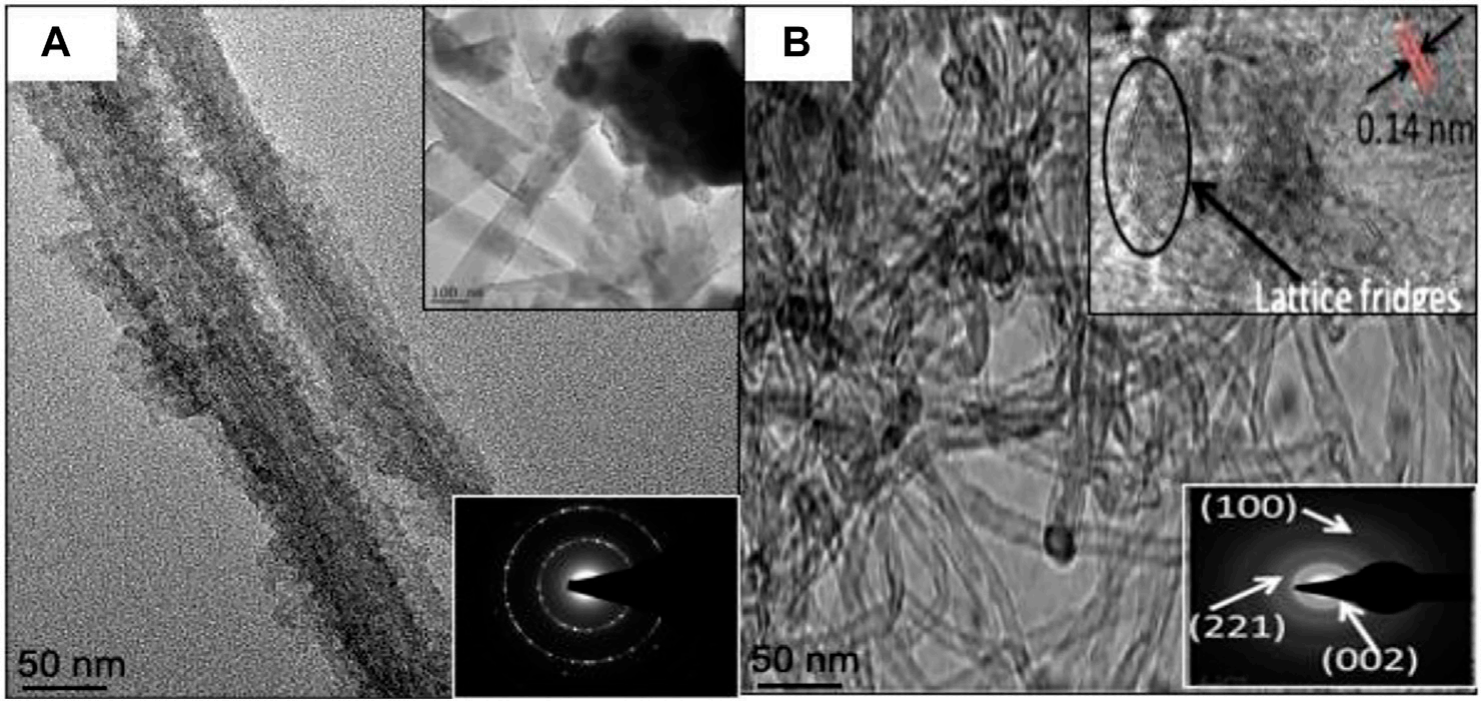
Transmission electron micrograph and Selected area (electron) diffraction (SAED) patterns of **(A)** C/LMO and **(B)** C/LFMPO.

High-resolution SEM (HRSEM) was used to probe the morphology of the as prepared C/LMO and C/LFMPO composite materials. The HRSEM micrograph shown in Figure 2A illustrates cubic-shaped, pure spinel LMO (Dushina et al., 2015) with average primary particles size of ∼100 nm. The aggregation of these primary particles formed secondary LMO particles which were randomly sized between a 100–200 nm range. Figure 2A inset confirms the elements in the parent compound. The C/LMO sample shown in Figure 2B displays well grown spinel features across the MWCNT morphology which is also evident from TEM in Figure 3A. The C/LMO micrograph shows ultrafine nanotubes with diameters that range from 50–100 nm and length of ∼2 μm (Wu et al., 2015). The high-magnification image demonstrates that the LMO powders were well attached in the nanotubes’ interspaces. Particle sizes do vary, but their distribution within the lattice is impressive. This nanoparticle attachment process is vital for achieving optimum accessibility of Li^+^ during lithiation/de-lithiation process (Xu et al., 2015).

The SEM image of LFMPO powders is shown in Figure 2C, with the inset confirming the respective elements present. The micrograph shows the powder consisting of spherically shaped pristine LFMPO with primary particles size of ∼50 nm. The agglomerated primary particles between 100–120 nm size range suggest well-grown olivine LFMPO crystals. Stranded carbon nanotubes with the LFMPO nanoparticles appearing along the MWCNT walls is revealed in Figure 2D. The interlayer spacing of MWCNTs is commonly considered equal or close to the interlayer spacing of graphene, 0.34 nm. The strands having a uniform diameter of 0.5–5 nm enlarges the surface area and facilitates effortless movement of Li^+^ during the lithiation/de-lithiation process within the 3D scaffold (Ma et al., 2022). After the addition of nanotubes, a significant change in appearance is clear. This is due to the residual carbon which plays an important role in hindering the growth and stabilizing of the composite material during heat treatment, which proves ideal in inhibiting manganese dissolution. The interlayers are controlled by the Mn−O−Fe interactions serving as an intermediate between the MWCNT and the LiPO_4_ matrix.

Figure 3 shows the TEM micrographs of C/LMO (a) and C/LFMPO (b) with well-developed lattice fringes observed for both samples. The clear lattice fringes indicate formation of pure material with high crystallites. In Figure 3A, the *d*-spacing at 8 Å of neighboring lattice fringes corresponds to the (111) plane of LMO. The highly ordered single crystalline nature of the particles is revealed by the well-defined (SAD) spots (inset). A moderately poly-dispersive C/LFMPO network with uniform diameter of 0.5–5 nm is seen in Figure 3B. The insert shows the high-resolution TEM (HRTEM) micrograph with spacing of Ca. 0.14 nm and in-plane (0–110) lattice, indicative of high crystallinity (Willenberg and Ross, 2020). This in turn contributes significantly to the Li^+^ diffusion through the electrolyte whilst structural integrity is maintained.

### Analysis of Phase Composition and Crystal Structure

**FIGURE 4:**
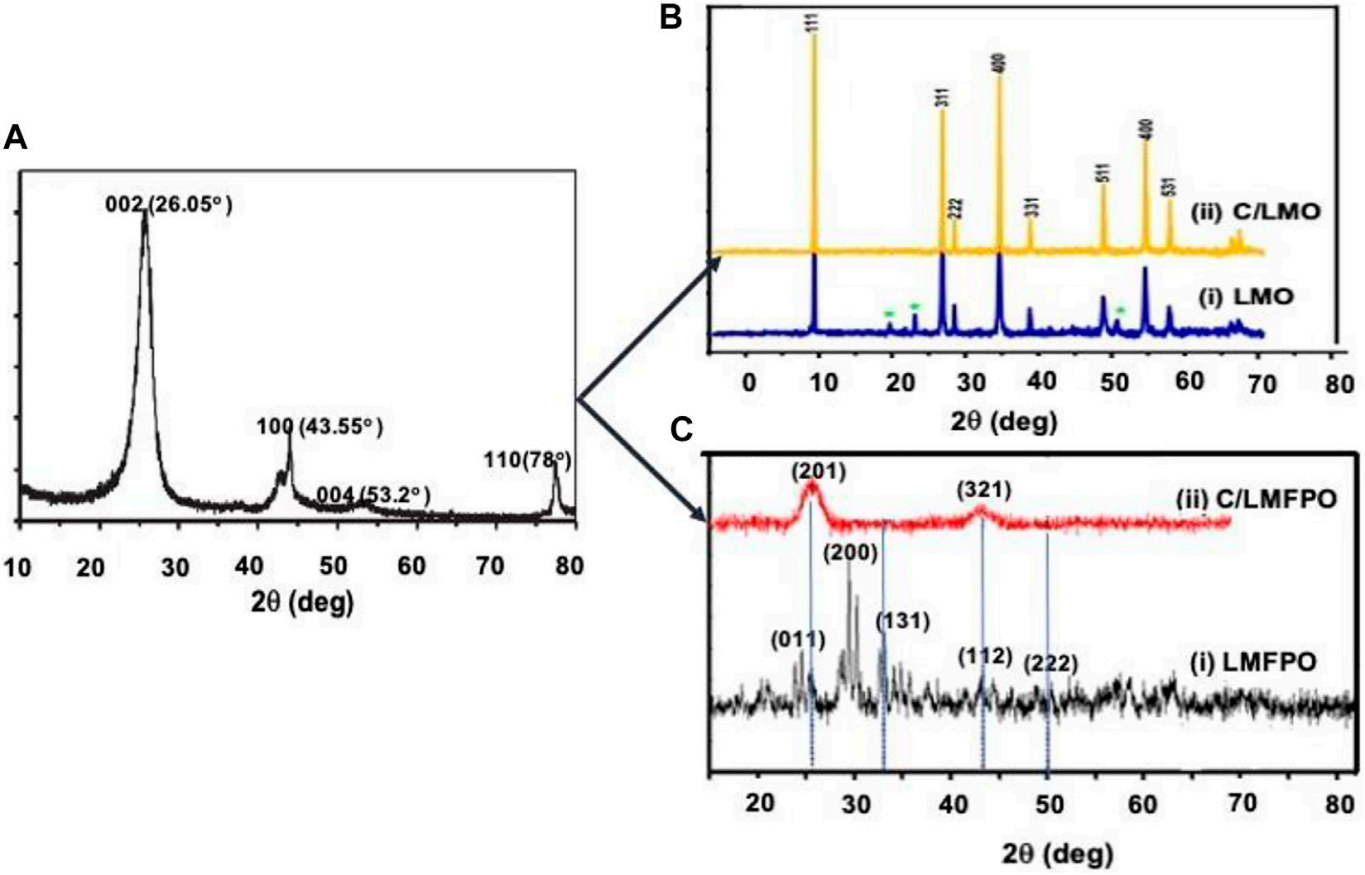
XRD graphs of **(A)** pure MWCNTs, **(B)** LMO(i) and C/LMO(ii), **(C)** LMFPO(i) and C/LMFPO(ii).

Figure 4 shows the XRD pattern of pure MWCNTs (a) LMO (i), C/LMO (ii) (b), LMFP (i) C/LFMP (ii) (c). The pattern of pure MWCNTs displays an intense diffraction peak around *2θ* = 26° and low intensity peaks around *2θ* = 44°, 53°, and 78° which corresponds to the representative graphite diffraction patterns at (002), (100), (004), and (110), respectively (Atchudan et al., 2015). In Figure 4B pure spinel phase LMO with a space group *Fd3m* is observed (Zhao et al., 2012) with no additional phases. The least-squares method used to calculate the lattice parameters from the diffraction data give a *d*-spacing value of 0.892 for LMO (i) and improved *d*-spacing of 0.32 nm for C/LMO (ii). This indicates that the inclusion of MWCNT in the lattice gives a more rigid spinel structure favorable towards decreasing Mn dissolution, which is confirmed by electrochemical analysis (Kumar et al., 2013). The XRD peaks shown in Figure 4C observed at *2θ* = 25.7°, 28.8°, and 32.7° correspond to (011), (200), and (131) crystal planes of LMFPO (i). This agrees with reported reflections (JCPDS 71-0636) (Yun et al., 2015). The peaks correlate well with an ordered olivine structure and a *Pnma* space group that can be indexed as a pure crystallized phase*.* The crystalline structure of C/LMFPO, (ii) shows two strong diffraction peaks at *2θ* = 26° and 43°, corresponding to (201) and (321) crystal planes which is indicative of crystalline and amorphous growth of carbon on LMFPO. The pure carbon nanotubes, indexed to (JCPDS No. 41-1487) (Manjunatha et al., 2012), are intense but, within the C/LMFPO composite, the peaks shift only slightly. The *d*-spacing values of about 0.14 nm correspond to 201 reflection in the C/LMFPO(ii) XRD pattern.

The Scherrer’s equation was applied to calculate the crystallized size of the prepared particles:

$$d=\frac{0.9\;\geq \lambda }{Bcos\theta }$$

The wavelength is symbolized by *λ*, the mean crystallite in volume by *d,* and the width at the maximum hump of the broadened diffraction peak is symbolized by *B* ^8^. The Scherrer equation analysis of the FWHM of the most intense peaks was collected and presented in Table 1.

**TABLE 1:** Scherrer equation peak analysis of the FWHM.

	d-spacing (nm)	Particle size (nm)
LMO	0.89	55 ± 1.942
C/LMO	0.32	60 ± 3.451
LMFPO	0.52	6 ± 1.752
C/LMFPO	0.14	3.7 ± 0.957

### X-Ray Photoemission Spectroscopy Results

XPS analysis provided information about the electronic structure of the C/LMO and C/LMFPO composites. The overall energy resolution (analyzer + x-ray source) of the spectra presented in this work was set to 0.6 eV for the high-resolution core level scans. Please note that the binding energy (BE) of the Li 1s core level overlaps with the broad multiple lines of the Mn 3p core level and it is therefore not included here (as it was not possible to extract the intrinsic line shape of the Li 1s core level from the superimposed line shapes).

**FIGURE 5:**
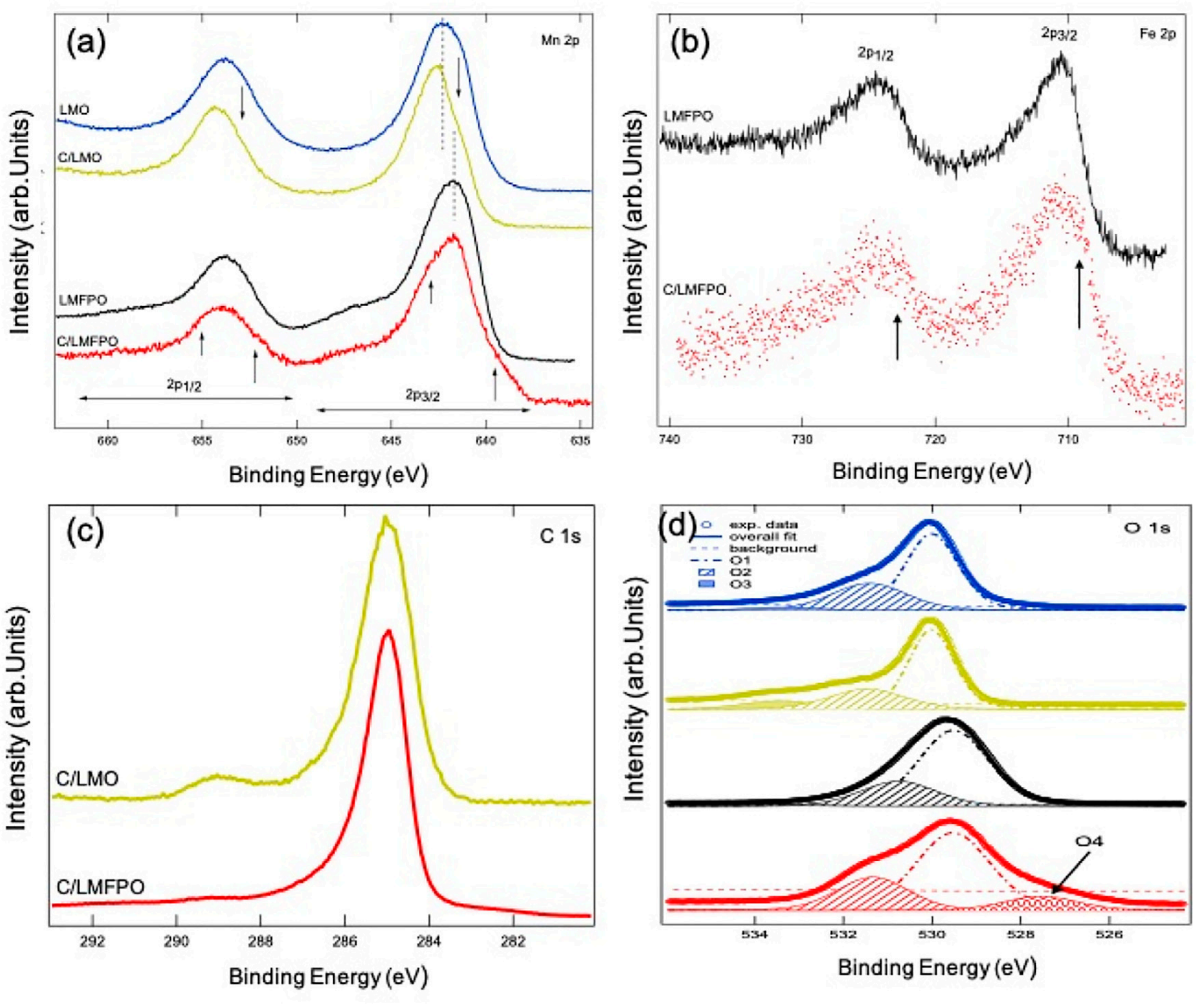
Mn 2p **(A)** and Fe 2p (**B)** core levels of the relevant composites, **(C)** shows the C 1s core level of C/LMO and C/LMFPO and **(D)** the O 1s core level spectra and fit results for the composites. The fit to the data (solod thick line) is superimposed to the experimental data with good agreement.

Figure 5 shows the 2p core levels of the 3d metal ions present in the composites. Figure 5A shows the Mn 2p binding energy (BE) region for all four composites. The Mn 2p core level is composed of two broad peaks whose centroids are located at BE’s of ∼641 and ∼653 eV5, corresponding to Mn 2p3/2 and Mn 2p1/2 spin orbit components, respectively. The Mn 2p core level line shape of LMO shows good correlation with that of Mn_2_O_3_ (Biesinger et al., 2011), having a majority 3^+^ oxidation state of Mn ions in this compound. The addition of carbon nanotubes to the LMO matrix causes the Mn 2p line shape to visibly change (yellow spectrum for C/LMO). This change is manifested in a suppression of the spectral weight on the lower BE side of the spin orbit peaks in C/LMO, as indicated by the two downward arrows in the figure. This suppression, as well as a shift of ∼0.5 eV towards higher BE of the main peaks, causes the line shape of the core level to look more similar to the one of MnO_2_ (or Mn^4+^) (Biesinger et al., 2011). It can therefore be inferred that CNTs alters the oxidation state of Mn ions from a majority 3^+^ in LMO to having a 4^+^ contribution in C/LMO.

We can compare the line shape of the Mn 2p core level of LMFPO and C/LMFPO in a similar fashion. Starting from LMFPO, we can see that 1) the centroid of the 2p_3/2_ spin orbit component has shifted to lower BE (∼640.3 eV), 2) the line shape of the peak is symmetric, and 3) a broad satellite in the BE region between 645 and 650 eV is now visible. The same features appear, although less evident, in the Mn 2p_1/2_ spin orbit component. All of these are the spectroscopic fingerprint of the fact that Mn ions in this composite have a majority 2^+^ oxidation state (Biesinger et al., 2011), as the line shape is comparable with that of MnO. Interestingly, the line shape for C/LMFPO shows changes that are worth discussing in detail. If we again focus on the 2p_3/2_ peak, we can see that 1) the intensity of the higher energy satellite decreases slightly and 2) the line shape of the main peak becomes less symmetric and shows a shoulder feature on the higher binding energy side of the main peak (see short upward arrow in the figure). These two features are compatible with a decrease in presence of Mn^2+^ and an increase in presence of Mn^3+^ in this compound. Furthermore, a prominent shoulder (indicated by upward arrows appears) appears on the lower BE side of the main peak. This very interesting spectral feature has been observed and documented in literature for manganites (Hishida et al., 2013). It is attributed to the appearance of well-screened Mn^3+^ ions due to an increase in metallicity of the samples, which, in this case, is brought about by the presence of carbon nanotubes.

Figure 5B shows the Fe 2p core level for LMFPO and C/LMFPO. Similarly to what we discussed above for Mn, the Fe 2p BE region is also composed of two (asymmetric) main peaks whose centroids are located at ∼711 and ∼725 eV, corresponding to Fe 2p_3/2_ and Fe 2p_1/2_ spin orbit components, respectively. Aside from the fact that the spectrum for C/LMFPO is more noisy due to decreased count rate, a noticeable difference in the line shape of the core level between the two composites is still detected. The spectrum for LMFPO shows a maximum of the intensity for the Fe 2p_3/2_ core level at ∼710.5 eV and its line shape is compatible with a majority 3^+^ oxidation state for Fe ions in this composite (Yamashita and Hayes, 2008). The addition of carbon nanotubes causes the appearance of extra spectral weight in the lower BE side of the peak, as indicated by the arrow in the figure. This is due to the contribution of Fe^2+^ in this composite (Yamashita and Hayes, 2008). Carbon nanotubes have the effect of shifting the oxidation state of Fe ions from being 3^+^ in LMFPO to being a mix 3^+^/2^+^ in C/LMFPO.

C 1s core level of C/LMO and C/LMFPO is reported in Figure 5C. The line shape of the two spectra shows very strong similarities and is consistent with what has been published in the literature with regards to MWCNTs (Varga et al., 2017). The main peak at ∼285 eV BE is due to C-C sp (Xinga et al., 2018) bonds, while the shoulder on its high BE side is due to sp (Willenberg and Ross, 2020) carbon bonds. The difference between the two spectra is that C/LMO shows a more pronounced peak at 289 eV BE, which is attributed to C=O bonds (Varga et al., 2017). This difference is attributed to the intrinsic microstructure of the composites.

Figure 5D shows the O 1s XPS spectra for the investigated samples. The spectra for LMO, C/LMO, and LMFPO have been fitted with three Voigt-type single components labelled as O1, O2, and O3 as shown in the figure (Dolla et al., 2018a). On the lower BE side of the spectrum, the O1 component is assigned to stoichiometric oxygen in the main matrix. The subsequent O2 can be assigned to oxygen vacancies or defects in the lattice (Dolla et al., 2018a) and O3 can be assigned to residual chemisorbed oxygen (i.e., surface contamination) (Dolla et al., 2018b). The fitted BE with the relative percentage area of each component is shown in Table 2. For the fitting of the O 1s core level spectrum of C/LMFPO, an extra component (O4) on the lower BE side of O1 had to be added to be able to reproduce the experimental results. This is consistent with what was reported in Figure 5A for the Mn 2p core level of this compound, and we can argue that O4 could be ascribed to the increased metallicity derived from the addition of MWCNTs.

**TABLE 2:** Fit results of the O 1s core level.

Sample	O1 (BE, eV)	O2 (BE, eV)	O3 (BE, eV)	O4 (BE, eV)	O1 area (%)	O2 area (%)	O3 area (%)	O4 area (%)
LMO	530.01	531.42	533.39	N/A	64	32	4	N/A
C/LMO	530.02	531.47	533.43	N/A	63	26.5	10.5	N/A
LMFPO	529.52	530.76	532.9	N/A	73.5	25.5	1	N/A
C/LMFPO	529.54	531.24	532.71	527.54	63	25.5	1	11.5

### Electrochemical Analysis

**FIGURE 6:**
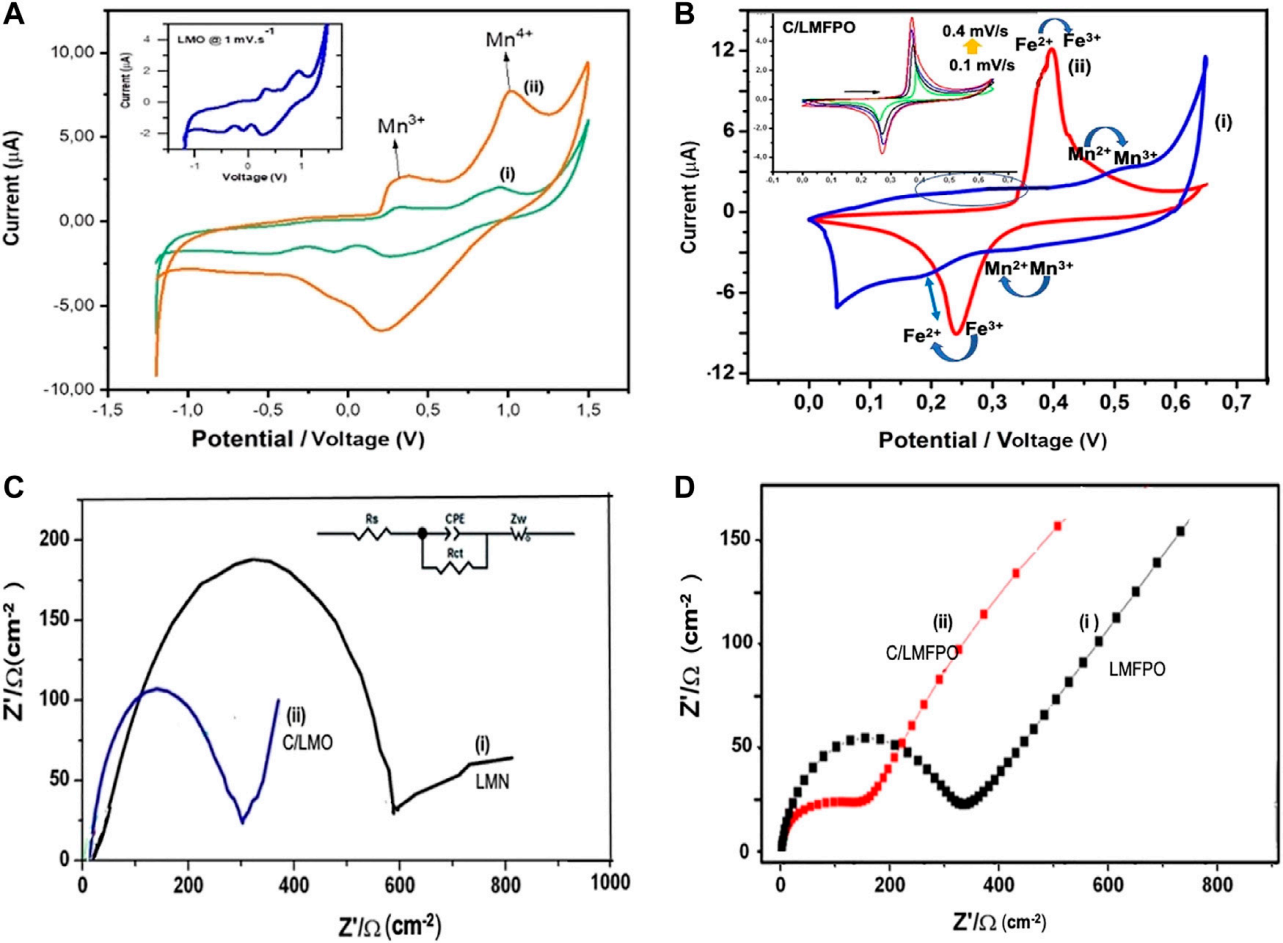
**(A,B)** Cyclic voltammograms of LMO (i) and C/LMO (ii) cycled at 1 mV s^−1^ with corresponding EIS analysis. **(C,D)** The Cyclic voltammograms of LMFPO(i) and C/LMFP(ii) in 1 M LiPF_6_ with corresponding EIS analysis.

Cyclic voltammetry (CV) and electrochemistry impendence (EIS) were used to probe the effect of MWCNT on the redox performance of C/LMO and C/LMFPO composites in aqueous media. The addition of carbon as conductive agent can prevent the irreversible phase transition often occurring in LMO and LMFPO systems (due to the high spin Mn^3+^) and electrolyte decomposition (Tang et al., 2015). Figure 6A shows the CV of the as-synthesized LMO (i) and C/LMO (ii) measured galvanostatically a scan rate of 0.1 mV s^−1^ in the potential range −1.3 to **+**1.5 V vs. Ag/AgCl^+^ in a LiPF_6_ solution. The redox peaks correlates to the surface oxidation/reduction of Mn^2+^/Mn^3+^ due to the charge transfer across the electrode/electrolyte interface (West et al., 2013). During the redox process, the Mn^3+/4+^ facilitates reversible Li^+^ diffusion reactions between electrodes. This process is promoted in an aqueous solution where Li^+^ drives the reaction (3) to the left, producing a stable environment for the intercalation compound (Willenberg and Ross, 2020).

LixA2B4 + xH2O ↔ A2B4 (s)+xLiOH (aq)+ x2 H2 (g)       (3)

The increase in the C/LMO electrode peak currents (ii) can also be attributed to well dispersed nanoparticles on the carbon surface, as shown by the SEM characterization. The connection along one-dimensional conduction pathways utilizes a larger surface area and enhanced electric conductivity, which result in improved reaction kinetics (Shen et al., 2019). The redox peaks peak potential difference (ΔE_p_) in the C/LMO composite shows a decrease from 0.77 V in LMP to 0.72 V, which suggests reduced polarization with improved electrochemical reversibility. Electrochemical impedance spectroscopy (EIS) was performed to investigate the effect of MWCNT on Li^+^ migration activity as well as interfacial properties. The LMO and C/LMO Nyquist plots in Figure 6B shows a well-defined single semi-circle at high frequency and an inclined line at low frequency attributed to Li^+^ diffusion in the bulk of electrode (Warburg impedance) (Vadhva et al., 2021). The equivalent circuit shown in Figure 6 inset was applied to simulate the experimental impedance spectra. The circuit consists of the following elements: charge transfer resistance (R_ct_), a CPE corresponding to double layer capacitance (Q_dl_), a Warburg element (Z_W_), and a solution resistance (R_s_) element (Xinga et al., 2018). The diameter of the semicircle relates to the charge transfer resistance within the composite (R_ct_). For LMO and C/LMFPO, the R_ct_ values were 226.3 and 187.1 Ω respectively. C/LMFP exhibits the least resistance to charge transfer due to having the smallest electrode polarization. The presence of conductive carbon in the composite decreases the resistance of system; hence, there is an increase in the current and broadening of the peak which contributes to the charge transfer capacitance.

Figure 6C shows the cyclic voltametric profile of LMFP (i) and C/LMFP (ii). Due to the collective Mn−O−Fe interactions in the olivine lattice, two separate redox centers corresponding to Fe^2+^/Fe^3+^ and Mn^2+^/Mn^3+^ are observed for LMFP (i). The respective redox activities corresponds to the electrochemical Li^+^ deinsertion/insertion upon the two phase oxidation/reduction process at ∼ 0.55/0.35 and 0.28/0.21 V, respectively.

The shape of the voltammogram in aqueous media (i) exhibits diminished and broadened peaks, indicative of a rather sluggish Li^+^ insertion/deinsertion behaviour (Ding et al., 2013) with a peak-to-peak separation ΔE_p_ = 1.21 V. The average LMFPO metal−oxygen bond length decrease and higher electronegativity of Fe depletes the ionic character of the Mn−O bond (Hyeon Kwon and Kim, 2019). The C/LMFPO composite (ii) shows more distinct peaks and a large, enclosed area with increased current, as well as reduced peak to peak separation ΔE_p_ = 0.21 V, at a similar scan rate. The C/LMFPO composite therefore displays better reversibility due to the carbon support on the crystal lattice. The formation of a stable 3D network facilities easier Li^+^ transport as it shortens the diffusion channel. The carbon coating makes the contact between particles more effective, thus improving the electron/ion conductivity (Chen et al., 2022). From Figure 6C inset, a linear relationship is observed which is indicative of a stable reversible electron transfer which is diffusion controlled. This was confirmed by electrochemical impedance spectroscopy analysis. Figure 6D shows the Nyquist plots for LMFPO(i) and C/LMFPO (ii) each having a well-defined single semi-circle. When studying the Nyquist plots in Figure 6D (ii) and (ii), the C/LMFPO shows a reduced resistance even compared to C/LMO.

Since both cathode materials have a similar nanocrystal size and amounts of carbon (2 mg), the decrease in R_ct_ can be attributed to the Fe. The Fe substitution compliments the LFMPO surface with more reactive Fe^3+/2+^ and Mn^4+/3+^ redox centers than in pristine LMPO. This corroborates with the XPS results. The enhancement of Li^+^ diffusion was confirmed by the time constant value which is an indication of the electron transfer kinetics and was calculated using: $\tau =\frac{1}{\omega_{max}}$ . Here an increase in τ correlates with a resistance of Li^+^ insertion and extraction due to an obstruction to the flow of the electrolyte at the interface. The ω_max_ is the angular frequency at the maximum impedance. The rate of current flow at the surface of the electrode at equilibrium was calculated using: $i_{o}=nFAKC.$ Here *n* represents the number of electrons transmitted per Li molecule = 1, *F* is the Faraday’s constant = 96485 C/mol, *A* is the geometric area of the electrode = 2.01 cm^2^, and *C* is the concentration of Li^+^.

The calculated values are shown in Table 3.

**TABLE 3:** The kinetic parameters of modified and unmodified samples done at 298 K.

	LMO	LFMPO	C/LMO	C/LFMPO
R_ct_/Ω	560.4	407.6	226.3	138.0
τ/s/rad	3.41 × 10^−4^	2.35 × 10^−4^	3.02 × 10^−4^	1.21 × 10^−5^
i_o_/A cm^−2^	1.80 × 10^−4^	6.30 × 10^−4^	2.75 × 10^–3^	1.86 × 10^−4^

According to the values in Table 3, it can be concluded that the C/LFMPO**-**composite has the lowest R_ct_, a more enhanced rate of change transfer (i_o_) due to reduced blocking of electrolyte at the electrode/electrolyte interface, and a decrease in time constant value( τ ) (Benbow et al., 2011). The faster kinetics can be attributed to the unique synergy between the conductive MWCNTs and the contribution of both single-phase and two-phase regions in Li_1-x_(Fe,Mn)PO_4_ during Li^+^ extraction and insertion. Essentially an improvement in the structural stability and electrochemistry was achieved in aqueous media^41^.

## Conclusion

The C/LMO and C/LMFPO composites were successfully prepared *via* a facile microwave-assisted two-step procedure and their redox chemistry in aqueous media was compared. The addition of carbon proved ideal in enhancing electrode stability and lowering the external resistance to enhance electron transport. The composition of nanocrystalline phase MWCNTs coated LFMPO composites were confirmed through XRD and XPS analysis. Electrochemical and Spectroscopic investigation showed that the electronic properties and the structure of the host compounds are highly improved due to the formation of a more conductive network with MWCNTs. The charge/discharge capacities obtained from the integrated area under the anodic peak at the scan rate of 10 mV/s indicate that the C/LFMPO and C/LMO composite cathode exhibited a charge and discharge capacity of 259 mA h/g/177 mA h/g and 115 mA h/g/44 mA h/g respectively. Carbon facilitates enhanced Li^+^ diffusion kinetics due to unhindered charge transfer and shorter diffusion length whilst also reducing Mn^3+^ decomposition. Therefore, this work provided a simplistic and effective strategy to construct compatible nanocomposite Mn-based cathodes for safer Li-ion batteries suitable for next-generation portable electronics.

## Data Availability

The raw data supporting the conclusion of this article will be made available by the authors, without undue reservation.
